# Supplementary material for: Health-promoting text messages to patients with hypertension—A randomized controlled trial in Swedish primary healthcare
Source: PLoS One. 2025 Feb 12;20(2):e0314868. doi: 10.1371/journal.pone.0314868 (PMC11819501; doi:10.1371/journal.pone.0314868)
Supplement: S1 Data — (PDF) [file pone.0314868.s002.pdf]

Supplementary material

Measurements:

- Blood pressure: Mean BP was calculated from two measurements (mean of three readings if first and second reading differed by > 5 mmHg).
- Heart rate: Heart rate was measured in conjunction with BP measurements.
- Waist circumference: Waist circumference was measured between iliac crest and lowest rib in a standing up exhaled phase.

Blood samples:

- HbA1c was analysed using capillary electrophoresis (Capillarys 3 TERA Hemoglobin A1c Kit-program, Sebia, France).
- Lipid fractions were assessed using either a Cobas 701 instrument (Roche Diagnostics, Basel, Switzerland) or an Atellica CH 930 Analyzer (Siemens Healthineers, Erlagen, Germany) depending on local laboratories at each study site.
