# Supplementary material for: Health-promoting text messages to patients with hypertension—A randomized controlled trial in Swedish primary healthcare
Source: PLoS One. 2025 Feb 12;20(2):e0314868. doi: 10.1371/journal.pone.0314868 (PMC11819501; doi:10.1371/journal.pone.0314868)
Supplement: S1 File — (DOCX) [file pone.0314868.s003.docx]

English translation of first part of the project plan:

Project title (sv):

SMS-based lifestyle intervention in primary care for patients with hypertension: a randomized controlled trial

Swedish summary:

Previous studies have shown that interventions via text messages significantly increase adherence to medication and improve treatment outcomes of chronic diseases. However, there are no previous studies that have analyzed the effect of text messages with lifestyle advice to patients with hypertension in primary care with the aim of improving blood pressure and other cardiovascular risk factors.

The hypothesis is that text messages with lifestyle advice leads to increased awareness of the hypertension disease, increased adherence to medication, increased physical activity and weight loss, which in turn produces a blood pressure-lowering effect.

The purpose of the study is to evaluate the effect on blood pressure of lifestyle advice administered via regularly sent text messages to blood pressure patients in primary care. Furthermore, possible changes in cardiovascular risk factors, quality of life and self-rated health are investigated.

The study is designed as a randomized controlled clinical trial at health centers in four different counties in Sweden, with the aim of including a total of 400 patients. The patients who are included will be randomized to two different groups at each health center; an SMS group and a control group. Blood pressure, long-term blood sugar, blood lipids, BMI, waist circumference, as well as self-rated health and quality of life questionnaires will be measured at baseline and after six months of intervention. The participants' blood pressure medication is not affected by the study but is managed as usual by their regular doctor. This applies in both the intervention and control groups.

Patients randomized to the intervention group will receive four SMS messages per week sent to their phone. The SMS will include advice on diet and exercise, general information on cardiovascular health, as well as advice on tobacco use specifically for smokers.

If an SMS intervention with lifestyle advice can have an effect on blood pressure and/or other risk factors for cardiovascular disease, it is a side-effect-free and inexpensive supplement to conventional blood pressure treatment.

It could also be that the intervention, in addition to reducing the patients' cardiovascular risk, can increase their self-perceived health and quality of life.
