# Supplementary material for: Health-promoting text messages to patients with hypertension—A randomized controlled trial in Swedish primary healthcare
Source: PLoS One. 2025 Feb 12;20(2):e0314868. doi: 10.1371/journal.pone.0314868 (PMC11819501; doi:10.1371/journal.pone.0314868)
Supplement: S2 File — (DOC) [file pone.0314868.s004.doc]

# PROJEKTPLAN

# Projekttitel (eng):

**PUSH ME (Primary care USage of Health promoting Messages): A text message-based intervention in primary care patients with hypertension: a randomized controlled trial**

# Projekttitel (sv):

SMS-baserad livsstilsintervention i primärvården för patienter med högt blodtryck: en randomiserad kontrollerad studie

**Svensk sammanfattning:**

Tidigare studier har visat att interventioner via SMS-meddelanden signifikant ökar följsamheten till medicinering och förbättrar behandlingsresultat av kroniska sjukdomar. Det finns dock inga tidigare studier som har analyserat effekten av SMS-meddelanden med livsstilsråd till patienter med högt blodtryck i primärvården med syftet att förbättra blodtryck och andra kardiovaskulära riskfaktorer.

Hypotesen är att SMS med livsstilsråd leder till ökad medvetenhet om hypertonisjukdomen, ökad följsamhet till medicinering, ökad fysisk aktivitet och viktnedgång, vilket i sin tur ger en blodtryckssänkande effekt.

Studiens syfte är att utvärdera effekten på blodtrycket av livsstilråd administrerade via regelbundet skickade SMS till blodtryckspatienter i primärvården. Vidare undersöks eventuella förändringar i kardiovaskulära riskfaktorer, livskvalitet och självskattad hälsa.

Studien är designad som en randomiserad kontrollerad klinisk studie på vårdcentraler i fyra olika landsting i Sverige, med mål att inkludera totalt 400 patienter. Patienterna som inkluderas kommer att randomiseras till två olika grupper på varje vårdcentral; en SMS-grupp och en kontrollgrupp. Blodtryck, långtidsblodsocker, blodfetter, BMI, midjeomfång samt frågeformulär om självskattad hälsa och livskvalitet kommer att mätas vid baslinjekontrollen och efter sex månaders intervention. Deltagarnas blodtrycksmedicinering påverkas inte av studien utan sköts som vanligt av deras ordinarie läkare. Detta gäller i både interventions- och kontrollgruppen.

De patienter som randomiseras till interventionsgruppen kommer att få fyra SMS per vecka skickade till sin telefon. SMS-en kommer att innehålla råd avseende kost och motion, allmän information om kardiovaskulär hälsa, samt råd om tobaksanvändning specifikt till rökare.

Om en SMS-intervention med livstilsråd kan ge effekt på blodtryck och/eller andra riskfaktorer för hjärtkärlsjukdom är det ett biverkningsfritt och billigt komplement till konventionell blodtrycksbehandling.

En förhoppning är också att interventionen, utöver att minska patienternas kardiovaskulära risk, kan öka deras självupplevda hälsa och livskvalitet.

## Background

The globalization of unhealthy lifestyles and demographic aging of the world’s population has contributed to the fact that high blood pressure (BP) is classified by the World Health Organization as the world’s leading risk for mortality. Although most high-income countries have had favorable trends with decreasing prevalence of high BP, a global increased prevalence of raised BP has been shown in a recent large meta-analysis . Hypertension is a key risk factor for cardiovascular diseases (CVD) . Modifiable lifestyle risk factors associated with hypertension, including smoking, unhealthy diet and physical inactivity, account for approximately 80% of CVD . About 30-40% of individuals with hypertension also have additional metabolic risk factors, such as dyslipidemia, insulin resistance and elevated blood-glucose , which further multiplies the risk for CVD . In most cases, lifestyle intervention can reverse or reduce the unfavorable metabolic profile .

Primary healthcare centers (PHCCs) in Sweden handle the majority of patients with hypertension. However, there are limited resources to intervene with the lifestyle changes needed for primary and secondary prevention of CVD and a large proportion of individuals with hypertension are uncontrolled or unaware of their condition .

Interventions by SMS have been shown to significantly improve compliance to medications, follow-up rate and disease monitoring . A Swedish study, which used an interactive mobile phone intervention on BP, showed improved BP control by self-management of hypertension . A randomized controlled trial (RCT) of CVD patients in Australia, who got weekly lifestyle focused SMS messages during six months, showed positive effects on both BP, body mass index (BMI), physical activity and smoking cessation , as compared to the control group. Thus, communication by telehealth, e.g. SMS (Short Message Services), could constitute an additional tool to reach patients for lifestyle advice. SMS is a common, convenient and cheap method of communication that can reach a large proportion of a primary care population.

Although positive results have been indicated for text messaging interventions for CVD risk factors, lifestyle-promoting messages have not been evaluated as a treatment for hypertension in primary care. We conducted a pilot study to evaluate the feasibility of an RCT with SMS intervention to promote lifestyle changes in individuals with hypertension in primary healthcare. The pilot study showed feasibility of the design and favorable trends regarding all included cardiovascular risk factors. Meanwhile, it is important to understand the factors contributing to an increased compliance to lifestyle interventions . Behavioral patterns can be predicted using the Theory of planned behavior (TPB) . According to the TPB can attitudes, perceived behavioral control and behavior intentions strongly predict individual behavior in a certain situation. TPB has for example been used to predict cardiovascular risk behavior in coronary patients , or to predict short-term weight loss in obese patients . TPB is therefore a useful model to understand, predict and model behavior.

## Objectives

The primary objective of the study is to examine the impact of lifestyle advices, administered through regularly sent SMS, on hypertension in a primary health care setting. The secondary objective is to evaluate changes in other cardiovascular risk factors and general health, e.g. tobacco use, obesity, blood lipids, HbA1c, self-rated health and health-related quality of life. Another secondary objective is to study the correlation between lifestyle changes and behavior predictors emerged from the TPB (attitudes, subjective norms, perceived behavioral control and behavioral intentions).

**Contribution to clinical praxis**

This project aims to investigate the use of e-health to assist health personnel in primary health care to carry out preventive measures of cardiovascular disease. As primary health care in Sweden is short of staff and overburdened with different assignments, life style counseling is not prioritized, although it is very important in the treatment of hypertension. If this study can show effect on blood pressure control, the text messages can be used as a complement to usual care and possibly through encouraging life style changes, prevent further cardiovascular disease. Understanding the relationship between behavioral change and individual behavior predictors might contribute to modeling tailored lifestyle interventions, in order to improve compliance and therefore increase the cost effectiveness.

# PROJECT DESCRIPTION

## Trial Design

Randomised controlled multi-centre study.

## Setting and participants

The study will involve 400 patients from 8 PHCCs located in four different regions in Sweden. Each PHCC in Sweden has a defined population of patients and several different professionals, e.g physicians, nurses, physiotherapists, psychologists, social workers and dieticians. The population of the PHCCs may differ by age distribution, morbidity and socioeconomic status.

### Inclusion Criteria

1. Patients with hypertension (defined by the International classification of disease Manual ICD-10, diagnose code I10.9)
2. 40-85 years
3. Patient must own a smart mobile phone

### Exclusion Criteria

1. Blood pressure at baseline visit ≥180/110 mmHg or systolic blood pressure <120 mmHg
2. Serious illness with short life expectancy (<1 year)
3. Predicted inability to comply with the study protocol e.g. language difficulties, interpreter needs, serious cognitive impairment

## Intervention

### Experimental treatment

The experimental treatment is an intervention addressing metabolic risk factors associated with cardiovascular disorders in patients with hypertension.

The intervention will consist of regularly delivered SMS messages aiming to remind, encourage and motivate patients to pursue healthy lifestyle changes. After baseline measurement, participants in the intervention group will receive four semi-personalized SMS messages per week for six months, in addition to their usual anti-hypertensive treatment. Each week, the participants will receive SMS from each of the following groups: A. Physical activity, B. Tobacco use, C. Dietary habits, and D. Cardiovascular health in general, except for non-smokers who, instead of the tobacco use-SMS, will get one extra randomly selected SMS from group A, C or D. The messages will be sent at random times during daytime between 9 AM and 7 PM. The SMS messages are developed by the authors using lifestyle recommendations based on Swedish national guidelines , and edited by an expert group at the Centre for lifestyle habits in Malmö including physiotherapists, dieticians and a physician specially trained in encouraging healthy lifestyle habits. Some SMS messages will contain links to educational material about life style habits and other SMS messages will serve as pure reminders of a healthy life style.

### Control Treatment

The control group will receive usual care according to the PHCCs usual practice.

## Endpoints

### Primary Endpoint

Change in blood pressure (mmHg)

### Secondary Endpoint

Changes in:

1. Cholesterol (total cholesterol, high-density lipoprotein [HDL], low-density lipoprotein [LDL]) (mmol/l)
2. Tobacco and alcohol use
3. BMI (kg/m^2^) /waist hip ratio
4. HbA1c (mmol/mol)
5. Self-rated health (five-graded Likert scale)
6. Health related quality of life, as measured by EQ5D-5L
7. Self-reported physical activity.

### Recruitment

Participants with hypertension will be identified through registers at the PHCC. Letters with information about the study will be sent to a random selection of all patients 40-85 years old with hypertension. If he patient chooses to participate, by answering positively to the information letter, they will be contacted by phone by a research assistant for additional information, possibility to ask questions about the study, and scheduling a baseline control at their PHCC.

### Baseline examination

Included patients that consent to take part in the study will be invited to their PHCC for a baseline visit. The following measurements will be assessed by a research assistant: blood pressure (in sitting position after 5 minutes rest; mean of two measurements in a standardized procedure with validated electronic BP devices), BMI and waist-hip circumference. Furthermore, the patients will complete a short questionnaire for evaluation of medical history, medication, tobacco and alcohol use, physical activity level, self-rated health and health-related quality of life. Blood samples for HbA1c and cholesterol will be drawn in the morning within a few days after the baseline visit.

Exclusion criteria include BP measurements at baseline control outside the range 120-180 (systolic) or ≥110 mmHg (diastolic), that is below the definitions for optimal SBP or above those for grade 3 hypertension respectively. Our consideration regarding these limits was that it seemed unlikely to have any BP lowering effect on patients with SBP <120 mmHg. However, we often see DBP values <80 mmHg in our hypertensive patients.

Regarding the upper limit, >180/110 mmHg, physical activity is not recommended to patients with these BP levels .

### Randomization

Randomization will be performed after completion of baseline assessments and questionnaires. A computer-generated random number schedule with block sizes of four will then be prepared. To ensure allocation concealment, a collaborator outside of the research project will perform the randomization. Information about group affiliation will be delivered to the patients by postal mail. The research assistant, the patients’ primary care physicians, as well as the researchers will be blinded to group allocation. If the patients have questions or want to exit the study, they will be able to call a telephone number to a collaborator not involved in the data analysis.

### Follow up A follow up control will be performed after 6 months with the same assessments as at the baseline visit. All participants will receive a questionnaire with items developed according to the TPB, targeting behavior predictors (Appendix).

***Data analysis***

The power analysis indicates a sample size of 189 patients in each arm. The calculation is based on an assumed statistical power of 80%, a two-sided test, using a significant level of 5% with a difference of 4 mm Hg between the groups, a standard deviation of 13 mm Hg and a drop out rate of 15%. Data will be analyzed according to the intention-to-treat principle. Differences in mean change of end points between intervention and control groups will be calculated by ANCOVA, with baseline values as covariates. Correlation between behavioral change (smoke cessation, increased level of physical activity) and behavioral predictors emerged from the TPB will be analyzed with logistic regression analysis.

## Ethical Considerations

The study will be conducted in accordance with the protocol, applicable regulatory requirements and the ethical principles of the Declaration of Helsinki as adopted by the 18^th^ World Medical Assembly. An application to the Swedish Ethical Review Authority will be submitted. All patients will be informed, both in written form and orally about the study. An informed consent will be signed and collected before enrolment in the study. The patient may at any time withdraw from the study without having to give an explanation and without affecting the patient’s future healthcare.

If extreme measures are found at baseline or follow up visit, the patient’s physician will be informed.

## Time Table

### Pre-study phase

Sept 2019-May 2020

Recruiting participating PHCCs
Application to Swedish Ethical Review Authority

Informing and coordinating centres.

Further development of text messages.

### Study phase

Inclusion period May 2020 – Dec 2021

Follow up period Nov 2020 – June 2021

Analysis phase
August 2021- December 2021 data management, analyses, and study report. Publication in a peer reviewed scientific journal.

### Project group

Beata Borgström Bolmsjö, PhD, MD. Specialist in Family Medicine

Susanna Calling, Associate professor, MD Specialist in Family Medicine

Veronica Milos Nymberg, PhD, MD. Specialist in Family Medicine

Moa Wolff, PhD, MD. Specialist in Family Medicine

## References
